# Prevalence of Adverse Events in Mexico Using the Institute for Healthcare Improvement—Global Trigger Tool Method: A Retrospective Study

**DOI:** 10.1111/jep.70405

**Published:** 2026-03-19

**Authors:** Luis Meave Gutierrez‐Mendoza, Elizabeth Manias, Patricia Nicholson

**Affiliations:** ^1^ School of Nursing and Midwifery, Centre for Quality and Patient Safety Research, Institute for Health Transformation Deakin University Geelong Victoria Australia; ^2^ Department of Public Health, School of Medicine Universidad Autonoma de San Luis Potosi San Luis Potosí San Luis Potosí Mexico; ^3^ Faculty of Medicine, Nursing and Health Science, Monash Nursing and Midwifery Monash University Melbourne Victoria Australia; ^4^ School of Medicine, Faculty of Health Deakin University Geelong Victoria Australia

**Keywords:** adverse events, global trigger tool, hospitalised patients, medical record review, patient safety, prevalence

## Abstract

**Rationale:**

Globally, adverse events (AEs) are a major contributor to mortality, often arising from healthcare management rather than patients' underlying conditions.

**Aims and Objectives:**

This study aimed to estimate the prevalence of AEs in three different hospitals in central‐north Mexico using the Institute for Healthcare Improvement Global Trigger Tool (IHI‐GTT) method.

**Method:**

A retrospective review of hospital discharges (July 2022 to June 2023) was conducted in three hospitals using the IHI‐GTT method. Records of patients aged ≥ 18 years with hospital stays longer than 24 h were reviewed, with obstetric and paediatrics cases excluded. One experienced physician performed the two‐stages review process, with inter‐rater reliability assessed on 1% of records. Logistic regression analysis was used to identify factors associated with AEs.

**Results:**

Among 3354 discharges, 36.6% (*n* = 1227) experienced at least one adverse event (AE), corresponding to 72.79 AEs per 1000 patient‐days or 53.04 AEs per 100 admissions. Of these, 72.8% (*n* = 893) were preventable. The most frequent AEs were medication errors (23.6%), intravenous line errors (23.5%), hospital acquired infections (16.4%), and surgical (9.9%). The AEs contributed to the death of the patient in 14.6% (*n* = 179) of cases. Risk factors for AEs included hospital transfers (OR: 1.53; 95% CI: 1.25–1.87, *p* < 0.001), age ≥ 60 years (OR: 1.49; 95% CI: 1.22–1.83, *p* < 0.001), comorbidities (OR: 1.15; 95% CI: 1.08–1.23, *p* < 0.001), and longer hospital stay (OR: 1.13; 95% CI: 1.11–1.14, *p* < 0.001). Elective admissions showed a protective effect (OR: 0.77; 95% CI: 0.62–0.97, *p* = 0.027).

**Conclusion:**

The prevalence of AEs in Mexico, a middle‐income country, is higher than previously reported. These finding underscore a pressing public health challenge requiring targeted interventions.

## 1 Introduction

There is global interest in identifying the prevalence of adverse events (AEs) in hospitalised patients, as they represent a critical patient safety concern [1, 2, 3, 4]. According to the World Health Organization (WHO), AEs have been identified as the third leading cause of death in the United States and one of the top causes of mortality worldwide [5, 6]. The burden is even greater in middle‐ and low‐income countries, where up to 2% of hospital admissions may result in death [7]. Importantly, these events arise from healthcare management rather than patients' underlying conditions [1, 7, 8, 9]. Healthcare settings have been described as one of the most unsafe environments in the world, with the risk of death in some settings exceeding that of a front‐line soldier [10]. These figures underscore the critical role of healthcare professionals, hospital administrators, policymakers and patient safety advocates in implementing strategies to minimise patients' harm.

Retrospective medical records reviews have been shown to be more accurate than voluntary reporting systems and patient safety indicators for identifying the prevalence of AEs in different healthcare settings [3, 11]. The two most widely used methods are the Harvard Medical Practice Study (HMPS) method, which employs 18 triggers, and the Institute for Healthcare Improvement Global Trigger Tool (IHI‐GTT) method, which incorporates 53 triggers [12]. Both methods have been validated in diverse healthcare contexts and are considered reliable for detecting AEs, enabling consistent measurements and supporting their use in research and quality improvement initiatives [3].

The HMPS method was first applied in 1984 in the United States to estimate the prevalence and incidence of AEs [1]. Since then, it has been replicated in multiple countries, including Australia [8], Brazil [13, 14], Canada [9], Denmark [15], Ireland [16], Portugal [17], Spain [18], Egypt, Jordania, Kenya, Morocco, Tunisia, Sudan, South Africa and Yemen [7]. The Institute for Healthcare Improvement developed the Global Trigger Tool method in 2003, and updated it in 2009, to strengthen AEs detection [12]. The IHI‐GTT method employs a two‐stage review: in the first stage, typically a nurse screens medical records for triggers; in the second, a physician reviews those positive triggers to confirm the presence of an adverse event (AE). Notably, this process can be conducted by a single experienced reviewer, which is particularly advantageous in resource‐limited settings [19, 20, 21, 22, 23]. Evidence also suggest that one experienced reviewer can identify more AEs than a team, with reliable and consistent outcomes [19, 20, 21, 22, 23, 24]. The IHI‐GTT method has been replicated in diverse countries including Argentina [25], Denmark [15], Italy [26], Norway [27, 28], Palestinian [29], South Korea [30], Spain [20], Sweden [28], Turkey [31] and the United States [11, 32]. Moreover, Norway and Sweden have implemented it as a routine practice in all hospitals [33]. Compared with voluntary reporting, the IHI‐GTT method detects up to 10 times more AEs and is 19 times more sensitive in their identification [11, 31].

To promote safer patient care worldwide, the WHO established the World Alliance for Patient Safety [34]. However, the evidence on the prevalence of AEs largely originates from high‐income countries, while data from middle‐ and low‐income countries remain limited [7]. Furthermore, the heterogeneity of existing studies continues to hinder accurate estimation of the magnitude of the problem. This heterogeneity has posed a significant challenge in accurately assessing the true scale of the problem.

In Mexico, the Official Actions for Patient Safety became mandatory for all healthcare institutions in September 2017. These actions align with International Patient Safety Goals of the Joint Commission International [35] and the WHO Global Patient Safety Action Plan 2021–2030 [36]. Within this context, the aim of this study was to explore the prevalence of AEs in three different hospitals in central‐north Mexico using the IHI‐GTT method. For this study, an AE was defined according to the IHI‐GTT method as ‘an unintended physical injury resulting from or contributed to by medical care that requires additional monitoring, treatment or hospitalisation, or results in death’ [12].

## 2 Materials and Methods

A retrospective medical records review study was conducted using the IHI‐GTT method [12]. The study is reported in accordance with the Strengthening the Reporting of Observational studies in Epidemiology (STROBE) guidelines for cross‐sectional studies [37] (Supporting Information S1: Table 1).

### 2.1 Setting

This study was conducted in the state of San Luis Potosi, central‐north Mexico in three hospitals, selected to represent the diversity of healthcare institutions in the country. The hospitals included a public tertiary teaching hospital, (Hospital A [400 beds]), a public nonteaching hospital, (Hospital B [185 beds]), and a private hospital, (Hospital C [125 beds]) as they provide comprehensive care in medicine, surgery, orthopaedics, gynaecology, obstetrics and paediatrics. The hospitals are situated in the metropolitan area providing services to more than 1.1 million people. Both public hospitals provide services for patients without social security, while the private hospital provides services for patients with private medical insurance, private company workers, local state level government workers or union members. The public hospitals are accredited by the Mexican General Direction of Quality and Health Education, while the private hospital is certified by the Mexican General Health Council and the Canadian Health Council. The three hospitals have paper‐based medical records.

### 2.2 Sampling

Data from the three hospitals were collected from 1 July 2022 to 30 June 2023. A complete list of all discharges between these dates was provided by the three hospital's Health Information Department following ethics approval. In this study, the total number of discharges was used as some patients admitted in a given month were not discharged during the same period. In addition, monthly data on voluntary reported AEs were obtained from the Healthcare Quality Departments to assess seasonal patterns, including periods when residency programs commence or during holiday seasons. To obtain a representative sample of the population, 4 months (July and October 2022, January and April 2023) were selected to capture variations across the year rather than a random sample of 20 medical records per month. Although the IHI‐GTT method recommends random sampling to support rapid estimation of baseline rates, four predefined full months were included to allow a comprehensive assessment of all eligible discharges across hospitals. The months were distributed across the calendar year to reduce potential temporal or seasonal effects. The public teaching hospital had 10,053 total discharges with 4876 discharges included in the medical record review (Supporting Information S2: Table 2). The public nonteaching hospital had 4905 total discharges with 2370 discharges included (Supporting Information S3: Table 3). The private hospital had 5831 total discharges with 3127 discharges included in the medical record review (Supporting Information S4: Table 4).

Patients aged 18 years or older with a hospitalisation of more than 24 h, and medical records that were complete with a discharge summary were included in the medical record review. Obstetric and paediatric patients were excluded because of the uniqueness of the care required. Records were assigned identification numbers for de‐identification, and all evidence of patient identification was removed from the extracted data.

### 2.3 Data Collection

The IHI‐GTT method has a collection data sheet that includes six modules: care, medication, surgical, intensive care, perinatal and emergency department. A two‐stage process following the IHI‐GTT method was implemented. Forty‐five triggers were used from the original 53 (Supporting Information S5: Table 5). A Qualtrics online research survey was created to collect the data. Demographic data such as age, sex, admission type, length of stay, total number of comorbidities and total number of transfers was also recorded.

### 2.4 Medical Record Review Process

The total number of discharges during the study period was obtained from the Medical Records Department at each hospital. Only complete medical records were eligible for inclusion. Medical records were reviewed using the two‐stage process described in the IHI‐GTT method. The review was conducted by the first author, a clinician with extensive training and experience in trigger‐based retrospective medical records review. In the first stage, all eligible medical records were screened using the 20‐min time limit recommended by the IHI‐GTT method to determine whether an adverse event had occurred. The medical records were reviewed in the following order: discharge summaries, medication orders, physician notes, nursing notes, operating theatre records (when applicable), laboratory results and other clinical documentation. In the second stage, medical records identified as positive for at least one adverse event underwent an in‐depth assessment by the same reviewer. This stage involved a detailed clinical evaluation and event classification. It is important to note that when a trigger was identified in stage 1 and the patient's outcome strongly suggested an AE had occurred, but no explicit documentation of harm was present in the medical or nursing notes, the case was classified as having no AE.

To minimise the risk of bias, 1% of medical records were independently assessed by a registered nurse. Despite the IHI‐GTT method not recommending measuring preventability, this information was collected. The severity was classified using the IHI‐GTT method classification, which ranged from category E to category I (Supporting Information S6: Table 6).

Ethics approval was received from the [removed for anonymity purposes] and the Ethics Board of the Ministry of Health in the State of San Luis Potosi, Mexico. Additionally, approval from local institutional review boards was obtained from all three participating hospitals.

### 2.5 Statistical Analysis

Descriptive statistics were used to summarise the data, with measures of central tendency and 95% confidence intervals reported where appropriate. The percentage of discharges with at least one AE, AEs per 1000 patient‐days, and AEs per 100 admissions were calculated.

For inferential analysis, a multivariable logistic regression models were used to evaluate the associations between patient characteristics (age, sex, admission type, length of stay, number of comorbidities, and number of hospital transfers) and the occurrence of AEs, both stratified by hospital and in the overall model. The general form of the model was: log (*P*(*Y* = 1)/(1‐*P*(*Y* = 1))) = *β*_0_ + *β*_1_ *X*_Age group_ + *β*_2_ *X*_Sex_ + *β*_3_ *X*_Admission type_ + *β*_4_ *X*_Length of stay_ + *β*_5_ *X*_Comorbidities_ + *β*_6_ *X*_Hospital transfers_, where *Y* = 1 indicates the occurrence of an AE. Age was categorised into 18–39, 40–59 and ≥ 60 years to capture clinically relevant life stages and to provide stable estimates within regression models. Adjusted odds ratios (aOR) with 95% confidence intervals were estimated, controlling for all covariates included in the model. Model calibration was assessed with the Hosmer‐Lemeshow goodness‐of‐fit test, grouping observations into deciles of predicted risk. No additional sensitivity analyses were performed, as the results remained stable across hospital‐specific and overall models. Data were compiled in Microsoft Excel (Microsoft Corp., Redmond, WA, USA) and analysed using Stata version 19 (StataCorp, College Station, TX, USA).

## 3 Results

### 3.1 Inter‐Rater Reliability

To ensure inter‐rater reliability, a random sample of 1% (*n* = 35) of medical records was independently reviewed by a registered nurse, with over 15 years of experience with the Mexican Evaluation Model of Integrated and Quality Medical Records, a framework used to assess the quality of medical records. Initial agreement was 85.7% (30/35). The Cohen's Kappa coefficient was 0.59, indicating moderate agreement. Following an in‐depth review, consensus was achieved in all cases, increasing agreement to 100% (35/35).

### 3.2 General Characteristics of the Sample

**Figure 1:**
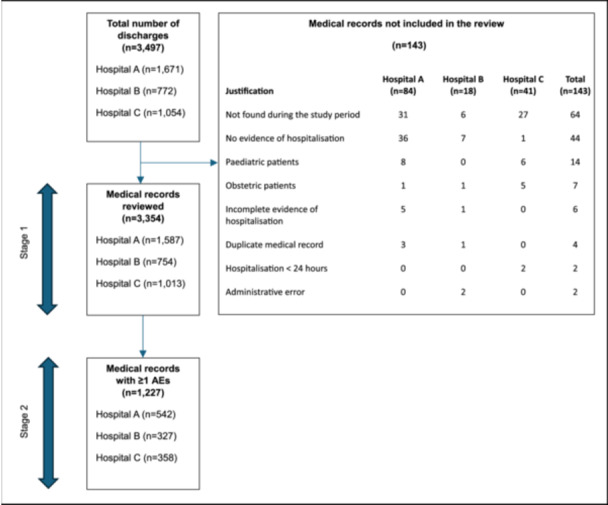
Model architecture design used in this study. The term ‘Adverse events’ is abbreviated to AEs.

A total of 3497 medical records were eligible for review across the three hospitals (Figure 1). Of these, 4.1% were not reviewed. The most frequent reasons for exclusion were medical records ‘not found during the study period’ and ‘no evidence of hospitalisation’, which together accounted for more than 75% of all exclusions. Detailed reasons for the excluded medical records are presented in Table 1.

**Table 1:** Medical records not included in the review.

	Hospital A	Hospital B	Hospital C	Total
Justification	*n* = 84 (%)	*n* = 18 (%)	*n* = 41 (%)	*n* = 143 (%)
Not found during the study period	31 (36.9)	6 (33.3)	27 (65.9)	64 (44.7)
No evidence of hospitalisation	36 (42.9)	7 (38.8)	1 (2.4)	44 (30.8)
Paediatric patients	8 (9.5)	0	6 (14.6)	14 (9.8)
Obstetric patients	1 (1.2)	1 (5.6)	5 (12.2)	7 (4.9)
Incomplete evidence of hospitalisation	5 (5.9)	1 (5.6)	0	6 (4.2)
Duplicate medical record	3 (3.6)	1 (5.6)	0	4 (2.8)
Hospitalisation < 24 h	0	0	2 (4.9)	2 (1.4)
Administrative error	0	2 (11.1)	0	2 (1.4)

Of the 3354 medical records, 52.1% (1746) corresponded to female patients and 47.9% (1608) to male patients. The median age of the patients who experienced an AE was 54 years [IQR 40–70] compared to 50 years [IQR 36–64] among those who did not. Most patients (81.6% [2736/3354]) were admitted via the emergency department, of whom 39.7% (1088/2736) experienced an AE, compared with 22.5% (139/618) of patients admitted electively. The median length of hospital stay was 7 days [IQR 4–13] for patients with an AE versus 4 days [IQR 2–6] for those without. The median number of comorbidities was 3 [IQR 2–4] among patients with an AE and 2 [IQR 1–3] among those without. The median number of hospital transfers was 1 [IQR 1–1] in both groups. Baseline characteristics of patients with and without AEs stratified by hospital are presented in Table 2.

**Table 2:** Baseline characteristics of patients with and without adverse events (AEs) by hospital.

Variable	Hospital A AE^a^ Yes (*n* = 542)	AE^a^ No (*n* = 1045)	Hospital B AE^a^ Yes (*n* = 327)	AE^a^ No (*n* = 427)	Hospital C AE^a^ Yes (*n* = 358)	AE^a^ No (*n* = 655)	Total AE^a^ Yes (*n* = 1227)	AE^a^ No (*n* = 2127)
Sex								
Male, *n* (%)	308 (38.6)	490 (61.4)	150 (40.5)	220 (59.5)	145 (33.0)	295 (67.0)	603 (37.5)	1005 (62.5)
Female, *n* (%)	234 (29.7)	555 (70.3)	177 (46.1)	207 (53.9)	213 (37.2)	360 (62.8)	624 (35.7)	1122 (64.3)
Age, median [IQR^b^]	49 [35–63]	47 [33–60]	57 [43–74]	51 [35–66]	60 [47–74]	52 [41–67]	54 [40–70]	50 [36–64]
18–39 years	30 [24–35]	28.5 [23–34]	30 [24–34]	28 [23–35]	32 [28–37]	31 [25–36]	31 [25–35]	29 [23–35]
40–59 years	49 [46–54]	50 [44–54]	49 [46–53]	50 [46–56]	51 [47–56]	49 [45–54]	50 [46–54]	49 [45–54]
≥ 60 years	71 [63.5–78]	70 [64–77]	74 [67–81]	71 [65–79]	73 [65–80]	71 [65–79]	73 [65–79]	71 [64–78]
Admission type								
Emergency *n* (%)	473 (87.3)	787 (75.3)	311 (95.1)	376 (88.0)	304 (84.9)	485 (74.0)	1088 (88.7)	1648 (77.5)
Elective *n* (%)	69 (12.7)	258 (24.7)	16 (4.9)	51 (12.0)	54 (15.1)	170 (26.0)	139 (11.3)	479 (22.5)
Length of stay, median [IQR^b^]	9 [5–18]	4 [2–7]	7 [4–12]	4 [3–7]	5 [3–8]	3 [2–4]	7 [4–13]	4 [2–6]
Comorbidities, median [IQR^b^]	3 [2–4]	2 [1–3]	3 [2–4]	2 [1–3]	3 [1–3]	2 [1–3]	3 [2–4]	2 [1–3]
Hospital transfers, median [IQR^b^]	1 [1–1]	1 [1–1]	1 [1–1]	1 [1–1]	1 [1–1]	1 [1–1]	1 [1–1]	1 [1–1]

*Note:* Sex and admission type are presented as *n* (%). Age, length of stay, comorbidities and hospital transfers are shown as median [IQR^b^].

a AE = adverse event.

b IQR = interquartile range.

### 3.3 The Occurrence of Adverse Events

The overall prevalence of discharges with at least one AE was 36.6% (1227/3354), ranging from 34.2% (542/1587) in Hospital A to 43.3% (327/754) in Hospital B. This corresponded to 72.8 AEs per 1000 patient‐days and 53.0 AEs per 100 admissions respectively. The rate per 1000 patient‐days accounts for both the number of patients and their length of stay, capturing the cumulative exposure to risk during hospitalisation; this measure indicates that Hospital C experienced the highest AEs burden relative to patient‐days. In contrast, the rate per 100 admissions standardised by the number of admissions regardless of stay duration, emphasised the proportion of patients experiencing AEs. Hospital B ranked highest, suggesting a greater proportion of discharges were associated with AEs, even though the average length of stay was shorter than in Hospital C (Table 3).

**Table 3:** Distribution, preventability and outcomes of adverse events (AEs) by hospital.

	Hospital A	Hospital B	Hospital C	Total
Adverse event (AE) characteristics	*n* = 1587 (%)	*n* = 754 (%)	*n* = 1013 (%)	*n* = 3354 (%)
Patients with ≥ 1 AE*	542 (34.2)	327 (43.3)	358 (35.3)	1227 (36.6)
AEs per 1000 patient‐days	57.5	86.9	103.2	72.8
AEs per 100 admissions	51.8	62.5	47.9	53.0
Preventable AEs	361 (66.6)	241 (73.7)	291 (81.3)	893 (72.8)
AEs related to healthcare management	500 (92.2)	315 (96.3)	347 (96.9)	1162 (94.7)
Total patient deaths**	146 (9.2)	89 (11.8)	31 (3.0)	266 (7.9)
Deaths in which an AE contributed***	96 (6.0)	62 (8.2)	21 (2.1)	179 (5.3)

*Note:* *Percentages for patients with ≥ 1 AE were calculated using the total number of medical records reviewed in stage 1 in each hospital as the denominator. Percentages for AEs are calculated using the total number of AEs identified in each hospital. **Total patient deaths include all in‐hospital mortality. ***Deaths in which an AE contributed represent a subset of total patient deaths and do not imply direct causation. The contribution of an AE to death was determined based on reviewer judgement following IHI‐GTT method guidance.

The proportion of preventable AEs was high across all hospitals, exceeding 66%, with the highest preventability observed in Hospital C (81.3%, 291/358). Most AEs (94.7%, 1162/1227) were determined to have a health management–related cause. General mortality among all discharges ranged from 3.1% (31/1013) to 11.8% (89/754), with an overall mortality of 5.3% (179/3354) attributable to an AE (Table 3).

Most admissions involved a single AE, accounting for 71.9% (882/1227) of all cases. Multiple AEs were less frequent, with two events in 17.4% (213/1227), three events in 6.9% (85/1227) and four or more events in 3.8% (47/1227) of discharges (Table 4).

**Table 4:** Total number of adverse events experienced per patient.

Number of adverse events (AEs)	Hospital A *n* = 542 (%)	Hospital B *n* = 327 (%)	Hospital C *n* = 358 (%)	Total *n* = 1227 (%)
1	385 (71.0)	224 (68.5)	273 (76.2)	882 (71.9)
2	83 (15.3)	72 (22.0)	58 (16.2)	213 (17.4)
3	46 (8.5)	23 (7.0)	16 (4.5)	85 (6.9)
4	13 (2.4)	7 (2.2)	8 (2.2)	28 (2.3)
5	12 (2.2)	0	2 (0.6)	14 (1.1)
6	1 (0.2)	1 (0.3)	1 (0.3)	3 (0.2)
7	0	0	0	0
8	2 (0.4)	0	0	2 (0.2)

When more than one AE was identified, categorisation was based on the most severe event. The most frequently identified categories across all hospitals were category E, with 42.9% (526/1227), and category F with 34.1% (419/1227), which together accounted for 77.0% (945/1227) of all events. Categories associated with more severe outcomes permanent harm (Category G), intervention to sustain life (Category H) and patient death (Category I), accounted for 1.3% (16/1227), 7.1% (87/1227) and 14.6% (179/1227) of AEs, respectively (Table 5).

**Table 5:** Categories of the 1227 patients that suffer at least one adverse event (AE) according to Institute for Healthcare Improvement Global Trigger Tool (IHI‐GTT) method.

	Hospital A	Hospital B	Hospital C	Total
Category	*n* = 542 (%)	*n* = 327 (%)	*n* = 358 (%)	*n* = 1227 (%)
E: Temporary harm to the patient and required intervention	167 (30.8)	166 (50.8)	193 (53.9)	526 (42.9)
F: Temporary harm to the patient and required initial or prolonged hospitalization	227 (41.9)	76 (23.2)	116 (32.4)	419 (34.1)
G: Permanent patient harm	8 (1.5)	5 (1.5)	3 (0.8)	16 (1.3)
H: Intervention required to sustain life	44 (8.1)	18 (5.5)	25 (7.0)	87 (7.1)
I: Patient death	96 (17.7)	62 (19.0)	21 (5.9)	179 (14.6)

The types of AEs were identified in the total number of identified AEs. Across all hospitals, the most frequent types of AEs were medication errors 23.7% (421/1779) and intravenous line errors 23.6% (419/1779), which together accounted for nearly half of all events (47.3%, 840/1,779). Hospital‐acquired infections and surgical events represented 16.4% (292/1779) and 10.0% (177/1779), respectively. Diagnostic errors (6.2%, 111/1,779), wrong procedure (6.2%, 111/1,779) and central venous line–related events (4.2%, 75/1779) together accounted for 16.6% of the total. Less frequent events included pressure injuries (2.2%, 40/1779), falls (0.9%, 16/1779) and other events (6.6%, 117/1779), such as resource limitations, technical complications or transfusion‐related reactions (Table 6).

**Table 6:** Type of the 1779 adverse events (AEs) identified in the 1227 patients.

	Hospital A	Hospital B	Hospital C	Total
Type of adverse event (AE)	*n* = 823 (%)	*n* = 471 (%)	*n* = 485 (%)	*n* = 1779 (%)
Medication error	200 (24.3)	106 (22.5)	115 (23.7)	421 (23.7)
Intravenous line errors	56 (6.8)	166 (35.2)	197 (40.6)	419 (23.6)
Hospital acquired infections	189 (22.9)	52 (11.0)	51 (10.5)	292 (16.4)
Surgical	124 (15.1)	26 (5.5)	27 (5.6)	177 (10.0)
Diagnostic errors	52 (6.3)	27 (5.8)	32 (6.6)	111 (6.2)
Wrong procedure	73 (8.9)	14 (3.0)	24 (5.0)	111 (6.2)
Central venous line related	41 (5.0)	27 (5.8)	7 (1.4)	75 (4.2)
Pressure ulcers	16 (1.9)	16 (3.4)	8 (1.7)	40 (2.2)
Falls	8 (1.0)	2 (0.4)	6 (1.2)	16 (0.9)
Others^a^	64 (7.8)	35 (7.4)	18 (3.7)	117 (6.6)

a Others is referring to: Resource‐related problems (e.g., lack of surgical materials, operating room or specialists physician; insufficient biopsy material), technical complications (e.g., accidental removal or need for relocation of pleural, urinary or central venous catheters; roof debris falling during surgery) and clinical outcomes (e.g., transfusion reactions, deep vein thrombosis, bronchoaspiration, haemorrhagic stroke after transfusion or cardiac perforation during stent placement).

Examples of ‘others’ AEs included resource‐related problems (e.g., lack of surgical materials, unavailability of an operating room or specialists physician; insufficient biopsy material), technical complications (e.g., accidental removal or need for relocation of pleural, urinary or central venous catheters; roof debris falling during surgery) and clinical outcomes (e.g., transfusion reactions, deep vein thrombosis, broncho‐aspiration, haemorrhagic stroke after transfusion, or cardiac perforation or pneumothorax during stent placement).

Based on univariate analyses of patient and healthcare utilisation characteristics, logistic regression was used to model the occurrence of AEs. In the overall model, patients aged ≥ 60 years had higher odds of experiencing an AE compared with those aged 18–39 years (aOR: 1.49, 95% CI: 1.22–1.83, *p* < 0.001). No significant association was observed between female sex and AE occurrence (aOR: 1.16, 95% CI: 0.99–1.36, *p* = 0.053). Elective admissions were associated with lower AE odds (aOR: 0.77, 95% CI: 0.62–0.97, *p* = 0.027). In addition, longer hospital stays (aOR: 1.13 per day, 95% CI: 1.11–1.14, *p* < 0.001), a greater number of comorbidities (aOR: 1.15, 95% CI: 1.08–1.23, *p* < 0.001), and more hospital transfers (aOR: 1.53, 95% CI: 1.25–1.87, *p *< 0.001) were all independently associated with higher AE risk. The model showed adequate calibration (Hosmer‐Lemeshow *χ* ^2^(8) = 9.70, *p* = 0.29) (Table 7).

**Table 7:** Multivariable logistic regression for factors associated with adverse events (AEs), by hospital.

	Hospital A (*n* = 1587)		Hospital B (*n* = 754)		Hospital C (*n* = 1013)		Total (*n* = 3354)	
Variable	aOR (95% CI)	*p* value	aOR (95% CI)	*p* value	aOR (95% CI)	*p* value	aOR (95% CI)	*p* value
Age group								
18–39	1.0	—	1.0	—	1.0	—	1.0	—
40–59	0.85 (0.64–1.14)	0.291	1.40 (0.90–2.16)	0.127	1.10 (0.73–1.66)	0.626	1.07 (0.87–1.31)	0.479
≥ 60	1.21 (0.90–1.63)	0.198	1.48 (0.96–2.26)	0.072	1.55 (1.01–2.37)	0.043	1.49 (1.22–1.83)	< 0.001
Sex								
Male	1.0	—	1.0	—	1.0	—	1.0	—
Female	0.87 (0.68–1.10)	0.260	1.45 (1.06–2.00)	0.020	1.41 (1.05–1.88)	0.019	1.16 (0.99–1.36)	0.053
Admission type								
Emergency	1.0	—	1.0	—	1.0	—	1.0	—
Elective	1.05 (0.76–1.46)	0.729	0.66 (0.35–1.24)	0.200	0.71 (0.49–1.03)	0.073	0.77 (0.62–0.97)	0.027
Length of stay (per day)	1.12 (1.10–1.14)	< 0.001	1.14 (1.10–1.18)	< 0.001	1.25 (1.19–1.32)	< 0.001	1.13 (1.11–1.14)	< 0.001
Comorbidities	1.19 (1.08–1.30)	< 0.001	1.27 (1.10–1.47)	< 0.001	1.03 (0.90–1.18)	0.611	1.15 (1.08–1.23)	< 0.001
Hospital transfers	1.57 (1.16–2.12)	0.003	1.29 (0.81–2.07)	0.272	1.46 (1.01–2.11)	0.042	1.53 (1.25–1.87)	< 0.001

*Note:* aOR adjusted for variables included in the model. Model fit: Hosmer‐Lemeshow *χ* ^2^(8) = 9.70, *p* = 0.29.

## 4 Discussion

To the best of our knowledge, this is the first study to estimate the prevalence of AEs in hospitalised patients using the IHI‐GTT method in Mexico. Our findings indicate that the IHI‐GTT method is a practical and reliable tool for assessing the prevalence of AEs, with results consistent across hospitals. Importantly, involving a smaller number of experienced reviewers in the medical record review process produced consistent outcomes, underscoring the importance of training and expertise in applying the tool. These results contribute new evidence from Latin America to the global literature on patient safety and support the feasibility of implementing structured trigger‐based methodologies in diverse healthcare systems.

This multicentre study found that more than one‐third of hospital discharges (36.6%, 1277/3354) were associated with at least one AE. Of these, 72.8% (893/1227) were deemed preventable, consistent with previous studies reporting preventability rates ranging from ≥ 60% [38] to 99% [13]. These findings highlight the substantial and potential avoidable burden of patient harm in Mexican hospitals, aligning with international estimates of AEs prevalence in Argentina [30%] [39], Brazil [33.7% and 40.9%] [14, 40], Italy [20.2%] [41] Portugal [36%] [42] and the United States [33.2%] [11]. In contrast, our results indicate a higher prevalence than previously reported in similar economies using the HMPS method, including 8.2% in the WHO Easter Mediterranean and African Regions [7], 10.5% in Ibero‐American countries (Argentina, Colombia, Costa Rica, Mexico, Peru) [38] and 15.7% (Brazil) [13]. The prevalence of AEs in this study highlights that they constitute a significant public health problem in middle‐income economies.

Regarding AEs severity, the most frequent categories identified in this study were E and F. These categories represent less severe forms of patient harm and are consistent with findings from previous studies in Italy [41], the United States [11], Denmark [15], Sweden [27], Switzerland [43] and Palestine [29]. The higher proportion of Category F events in the public teaching hospital likely reflects differences in patient case mix, as these institutions often manage more complex or severe conditions requiring prolonged hospitalisation. Conversely, the greater proportion of Category E events in the private and public non‐teaching hospitals may be related to differences in service delivery models, patient turnover, and intervention thresholds, where shorter lengths of stay and rapid access to interventions may result in more cases of temporary harm managed without extended hospitalisation. Specifically, Hospitals B and C showed higher proportions of Category E events, whereas Hospital A reported a greater proportion of Category F events.

The most frequent types of AEs in our study were medication errors and intravenous line errors, identified across all hospital types. This prevalence likely reflects the frequency of these procedures in routine care and their potential for harm in diverse clinical contexts. This finding adds to the existing literature, as intravenous line errors have not been previously reported as a leading cause of AEs in retrospective medical records review. The high frequency of intravenous line errors identified in this study differs from reports using trigger tool methodologies, in which medication errors, hospital acquired infections, or procedural complications were more commonly reported. Nevertheless, as reported in a recent systematic review, one in three (36.4%) peripheral catheters worldwide resulted in a complication, underscoring their importance as a source of patient harm [44]. The low frequency of intravenous line errors documented in hospital A may reflect contextual and organisational influences, including documentation practices. In addition, the quality of peripheral venous catheters, supply availability and nurse–patient ratios may have shaped the occurrence and detection of this type of AEs. Strengthening adherence to standardised protocols for peripheral venous catheter selection, insertion and maintenance, together with implementing care bundles, ensuring adequate nurse–patient ratios, and supporting staff through training and routine monitoring, could help reduce these adverse events. Hospital‐acquired infections and surgical error were the third and fourth most frequent types of AEs in our study. These results are partially consistent with a study conducted in two Brazilian teaching hospitals, where hospital acquired infection [47.1%] and surgical error [24.5%] were the most common AEs [13]. Medication errors have been reported as leading cause of AEs in the United States [11] and the second most common in Korea [30]. The higher proportion of hospital‐acquired infections and surgical events in the public teaching hospital in our study may be related to the complexity of cases managed, greater surgical volumes, and the training environment, which could introduce variability in procedural execution. In contrast, the elevated proportion of intravenous line errors in the non‐teaching and private hospitals may indicate differences in intravenous therapy protocols, staffing documenting patterns, the quality of catheter material, or resource allocation for catheter care.

We identified key patient‐ and admission‐related factors associated with AE occurrence, with notable variation by hospital type. Across the overall sample, older age (≥ 60 years), longer hospital stay, higher comorbidity burden, and more hospital transfers were associated with an increased risk of AEs. These findings align with previous research showing that patient age ≥ 60 years [45], patient complexity and prolonged hospitalisation elevate the likelihood of harm [30], whether through more frequent procedures, polypharmacy or cumulative risk of healthcare‐associated infections [11]. Conversely, elective admission was associated with lower odds of AEs, consistent with findings from Korea where a 3.73‐fold higher risk of AEs was observed among patients admitted through the emergency department [30]. This association underscore the role of acute illness severity and unplanned care pathways in influencing patient safety outcomes.

The variation in risk profiles across hospital types highlights the influence of institutional and contextual factors on AEs occurrence. For example, the association between female sex and higher AE risk observed in public non‐teaching and private hospitals may reflect differences in the types of services provided, diagnostic pathways, or documentation practices. Similarly, the association of length of stay in private hospitals could indicate differences in discharge practices, resource allocation or patient turnover. These findings suggest that institutional characteristics such as staffing models, case mix, clinical workflows and resource availability may not only affect the likelihood of AEs but also their detection. Understanding these patterns is critical for tailoring safety improvement strategies. Interventions targeting high‐risk patient groups, optimising transitions of care and reinforcing documentation practices could yield substantial reductions in AE rates that could benefit public and private health sector.

In the present study, 4.1% (*n* = 146) of medical records were not assessed for different reasons, which is lower than a previous study [14.2%] [7]. The reasons for excluding medical records in our study varied between hospitals, reflecting differences in record availability, documentation practices, and patient populations. High proportions of cases classified as ‘not found during the study period’ or ‘not evidence of hospitalisation’ suggest potential gaps in record retrieval systems or inconsistencies in hospital admission coding. This can be minimised when the hospital has an electronic medical record system [30].

### 4.1 Strengths and Limitations

Our study has several strengths that have not been addressed in previous studies. First, full months of discharges were reviewed rather than a random sample of medical records, allowing us to capture the real magnitude of the problem more accurately. Second, it is one of the largest multicentre evaluations of AEs in Latin America, encompassing diverse hospital types, public teaching, public non‐teaching, and private hospitals, thus enhancing the generalisability of our findings across different institutional contexts. Third, the use of the IHI‐GTT method, applied across all sites, ensures methodological comparability and supports robust cross‐hospital prevalence and severity analyses. The inclusion of both patient‐level and hospital‐level variables in the regression models allowed us to disentangle individual risk factors from system‐level influences on AEs occurrence and severity. Another strength is the involvement of an independent physician‐researcher not affiliated with the participating hospitals, enhancing the consistency and reliability of findings that can inform strategies to improve patient outcomes.

However, some limitations must be acknowledged. First, the retrospective design, inherent to trigger tool methodology relies on the completeness and accuracy of medical records; under‐documentation may have led to underestimation of AEs rates, particularly for events with subtle clinical manifestations or those resolved without formal documentation. Second, some medical records were not available for review in the three hospitals. The absence of consensus in the literature on acceptable predictive value thresholds for triggers also limits our ability to benchmark the tools performance against external standards. Finally, while our sample was large and diverse, the findings may not be directly applicable to healthcare systems with markedly different patient populations, structures or resources.

## 5 Conclusions

The high prevalence of AEs in Mexican hospitals constitutes a significant patient safety challenge that requires coordinated action from healthcare professionals, administrators, policymakers and other stakeholders to minimise patient harm. Similar issues may affect other high‐, middle‐ and low‐income countries where reliable measurement methods have not yet been applied. The IHI‐GTT method should be considered the preferred approach for hospitals seeking to estimate the prevalence of AEs, given it demonstrated reliability and adaptability. Ensuring the involvement of independent, experienced reviewers is essential in both public and private institutions. Moreover, using a census of at least 1 month of hospital discharges, rather than smaller samples, provides a more accurate estimate of the true magnitude of the problem. Finally, efforts to reduce AEs should prioritise safer prescribing practices to prevent medication interactions, stronger supervision of procedures and minimising unnecessary invasive interventions, rather than relying solely on additional resource allocation.

## Ethics Statement

Ethics approval was obtained from the Deakin University Human Research Ethics Committee (DUHREC 2023‐287), the Ministry of Health in Mexico (SLP/06‐2023) and the ethics committees of the participating hospitals.

## Conflicts of Interest

The authors declare no conflicts of interest.

## Supporting information


**Supplementary Table S1:** The STROBE reporting checklist.

**Supplementary Table S2:** Characteristics of hospital discharges in Hospital A.

**Supplementary Table S3:** Characteristics of hospital discharges in Hospital B.

**Supplementary Table S4:** Characteristics of hospital discharges in Hospital C.

**Supplementary Table S5:** Triggers used from the Institute for Healthcare Improvement Global Trigger Tool (IHI‐GTT) for Measuring Adverse Events (1).

**Supplementary Table S6:** Categories according to the Institute for Healthcare Improvement Global Trigger Tool (IHI‐GTT) (1).

## Data Availability

The data that support the findings of this study are available from the corresponding author, P.N., upon reasonable request. The data sets are not publicly available due to the presence of information that could compromise participant privacy or confidentiality.

## References

[1] T. A. Brennan , L. L. Leape , N. M. Laird , et al., “Incidence of Adverse Events and Negligence in Hospitalized Patients: Results of the Harvard Medical Practice Study I,” New England Journal of Medicine 324 (1991): 370–376, 10.1056/NEJM199102073240604. 1987460

[2] P. D. Hibbert , C. J. Molloy , T. J. Schultz , A. Carson‐Stevens , and J. Braithwaite , “Comparing Rates of Adverse Events Detected in Incident Reporting and the Global Trigger Tool: A Systematic Review,” International Journal for Quality in Health Care 35, no. 3 (2023): mzad056, 10.1093/intqhc/mzad056. 37440353 PMC10367579

[3] D. O. Klein , R. J. M. W. Rennenberg , R. P. Koopmans , and M. H. Prins , “A Systematic Review of Methods for Medical Record Analysis to Detect Adverse Events in Hospitalized Patients,” Journal of Patient Safety 17, no. 8 (2021): e1234–e1240, 10.1097/PTS.0000000000000670. 32168280 PMC8612912

[4] L. C. Eggenschwiler , A. W. S. Rutjes , S. N. Musy , et al., “Variation in Detected Adverse Events Using Trigger Tools: A Systematic Review and Meta‐Analysis,” PLoS One 17, no. 9 (2022): e0273800, 10.1371/journal.pone.0273800. 36048863 PMC9436152

[5] M. A. Makary and M. Daniel , “Medical Error—The Third Leading Cause of Death in the US,” BMJ 353 (2016): i2139, 10.1136/bmj.i2139. 27143499

[6] World Health Organization , Patient Safety, Global Action on Patient Safety, Report by the Director‐General (WHO, 2018). Report No.: EB144/29).

[7] R. M. Wilson , P. Michel , S. Olsen , et al., “Patient Safety in Developing Countries: Retrospective Estimation of Scale and Nature of Harm to Patients in Hospital,” BMJ 344 (2012): e832, 10.1136/bmj.e832. 22416061

[8] R. M. Wilson , W. B. Runciman , R. W. Gibberd , B. T. Harrison , L. Newby , and J. D. Hamilton , “The Quality in Australian Health Care Study,” Medical Journal of Australia 163, no. 9 (1995): 458–471, 10.5694/j.1326-5377.1995.tb124691.x. 7476634

[9] G. R. Baker , “The Canadian Adverse Events Study: The Incidence of Adverse Events Among Hospital Patients in Canada,” Canadian Medical Association Journal 170, no. 11 (2004): 1678–1686, 10.1503/cmaj.1040498. 15159366 PMC408508

[10] R. S. Barrett and L. H. Francescutti , “Why a Hospital Is the Most Dangerous Place on Earth,” in Hardwired: How Our Instincts to be Healthy Are Making us Sick (Springer International Publishing, 2021), 1–23, 10.1007/978-3-030-51729-8_1.

[11] D. C. Classen , R. Resar , F. Griffin , et al., “Global Trigger Tool' Shows That Adverse Events in Hospitals May Be Ten Times Greater Than Previously Measured,” Health Affairs 30, no. 4 (2011): 581–589, 10.1377/hlthaff.2011.0190. 21471476

[12] F. A. Griffin and R. K. Resar IHI Global Trigger Tool for Measuring Adverse Events (Second Edition). IHI Innovation Series White Paper (Institute for Healthcare Improvement; 2009), www.IHI.org.

[13] A. J. Lima Júnior , A. C. B. Zanetti , B. M. Dias , A. Bernardes , F. M. Gastaldi , and C. S. Gabriel , “Occurrence and Preventability of Adverse Events in Hospitals: A Retrospective Study,” Revista Brasileira de Enfermagem 76, no. 3 (2023): e20220025, 10.1590/0034-7167-2022-0025. 37436233 PMC10332367

[14] A. C. B. Zanetti , B. M. Dias , A. Bernardes , et al., “Incidence and Preventability of Adverse Events in Adult Patients Admitted to a Brazilian Teaching Hospital,” PLoS One 16, no. 4 (2021): e0249531, 10.1371/journal.pone.0249531. 33857137 PMC8049336

[15] C. Von Plessen , A. M. Kodal , and J. Anhøj , “Experiences With Global Trigger Tool Reviews in Five Danish Hospitals: An Implementation Study,” BMJ Open 2, no. 5 (2012): e001324, 10.1136/bmjopen-2012-001324. PMC3488702 23065451

[16] N. Rafter , A. Hickey , R. M. Conroy , et al., “The Irish National Adverse Events Study (INAES): The Frequency and Nature of Adverse Events in Irish Hospitals—A Retrospective Record Review Study,” BMJ Quality & Safety 26, no. 2 (2017): 111–119, 10.1136/bmjqs-2015-004828. PMC5284341 26862223

[17] P. Sousa , A. S. Uva , F. Serranheira , M. S. Uva , and C. Nunes , “Patient and Hospital Characteristics That Influence Incidence of Adverse Events in Acute Public Hospitals in Portugal: A Retrospective Cohort Study,” International Journal for Quality in Health Care 30, no. 2 (2018): 132–137, 10.1093/intqhc/mzx190. 29309608 PMC5890867

[18] J. L. Valencia‐Martín , J. Vicente‐Guijarro , D. San Jose‐Saras , P. Moreno‐Nunez , A. Pardo‐Hernández , and J. M. Aranaz‐Andrés , “Prevalence, Characteristics, and Impact of Adverse Events in 34 Madrid Hospitals. The ESHMAD Study,” European Journal of Clinical Investigation 52, no. 12 (2022): e13851, 10.1111/eci.13851. 35909351 PMC9787492

[19] R. Kaibel Val , P. Ruiz López , A. I. Pérez Zapata , A. Gómez de la Cámara , and F. de la Cruz Vigo , “Detection of Adverse Events in Thyroid and Parathyroid Surgery Using Trigger Tool and Minimum Basic Data Set (MBDS),” Journal of Healthcare Quality Research 35, no. 6 (2020): 348–354, 10.1016/j.jhqr.2020.08.001. 33115613

[20] O. Guzmán‐Ruiz , P. Ruiz‐López , A. Gómez‐Cámara , and M. Ramírez‐Martín , “Detección de eventos adversos en pacientes adultos hospitalizados mediante el método Global TriggerTool [Detection of Adverse Events in Hospitalized Adult Patients by Using the Global Trigger Tool method],” Revista de Calidad Asistencial 30, no. 4 (2015): 166–174, 10.1016/j.cali.2015.03.003. 26025386

[21] P. K. Pettersson , O. Sköldenberg , B. Samuelsson , A. Stark , O. Muren , and M. Unbeck , “The Identification of Adverse Events in Hip Fracture Patients Using the Global Trigger Tool: A Prospective Observational Cohort Study,” International Journal of Orthopaedic and Trauma Nursing 38 (2020): 100779, 10.1016/j.ijotn.2020.100779. 32439319

[22] B. D. Franklin , S. Birch , I. Savage , et al., “Methodological Variability in Detecting Prescribing Errors and Consequences for the Evaluation of Interventions,” Pharmacoepidemiology and Drug Safety 18, no. 11 (2009): 992–999, 10.1002/pds.1811. 19634116

[23] B. D. Franklin , S. Birch , M. Schachter , and N. Barber , “Testing a Trigger Tool as a Method of Detecting Harm From Medication Errors in a UK Hospital: A Pilot Study,” International Journal of Pharmacy Practice 18, no. 5 (2010): 305–311, 10.1111/j.2042-7174.2010.00058.x. 20840687

[24] M. Kobayashi , S. Ikeda , N. Kitazawa , and H. Sakai , “Validity of Retrospective Review of Medical Records as a Means of Identifying Adverse Events: Comparison Between Medical Records and Accident Reports,” Journal of Evaluation in Clinical Practice 14, no. 1 (2008): 126–130, 10.1111/j.1365-2753.2007.00818.x. 18211655

[25] A. Fajreldines , M. Pellizzari , M. Valerio , and V. Rodriguez , “Healthcare‐Associated Harm in Adult Patients Hospitalized in Two Tertiary Care Centers in Argentina,” Medicina 82, no. 3 (2022): 423–427. 35639064

[26] E. Scarpis , P. Cautero , A. Tullio , et al., “Are Adverse Events Related to the Completeness of Clinical Records? Results From a Retrospective Records Review Using the Global Trigger Tool,” International Journal for Quality in Health Care 35, no. 4 (2023): mzad094, 10.1093/intqhc/mzad094. 37952101

[27] H. Rutberg , M. Borgstedt Risberg , R. Sjödahl , P. Nordqvist , L. Valter , and L. Nilsson , “Characterisations of Adverse Events Detected in a University Hospital: A 4‐Year Study Using the Global Trigger Tool Method,” BMJ Open 4, no. 5 (2014): e004879, 10.1136/bmjopen-2014-004879. PMC4039822 24871538

[28] E. T. Deilkås , M. B. Risberg , M. Haugen , et al., “Exploring Similarities and Differences in Hospital Adverse Event Rates Between Norway and Sweden Using Global Trigger Tool,” BMJ Open 7, no. 3 (2017): e012492, 10.1136/bmjopen-2016-012492. PMC5372041 28320786

[29] S. Najjar , M. Hamdan , M. C. Euwema , et al., “The Global Trigger Tool Shows That One Out of Seven Patients Suffers Harm in Palestinian Hospitals: Challenges for Launching a Strategic Safety Plan,” International Journal for Quality in Health Care 25, no. 6 (2013): 640–647, 10.1093/intqhc/mzt066. 24141012

[30] J. I. Hwang , H. J. Chin , and Y. S. Chang , “Characteristics Associated With the Occurrence of Adverse Events: A Retrospective Medical Record Review Using the Global Trigger Tool in a Fully Digitalized Tertiary Teaching Hospital in Korea,” Journal of Evaluation in Clinical Practice 20, no. 1 (2014): 27–35, 10.1111/jep.12075. 23890097

[31] M. N. Kurutkan , E. Usta , F. Orhan , and M. C. E. Simsekler , “Application of the IHI Global Trigger Tool in Measuring the Adverse Event Rate in a Turkish Healthcare Setting,” International Journal of Risk & Safety in Medicine 27, no. 1 (2015): 11–21, 10.3233/JRS-150639. 25766063

[32] J. M. Naessens , T. J. O'Byrne , M. G. Johnson , M. B. Vansuch , C. M. McGlone , and J. M. Huddleston , “Measuring Hospital Adverse Events: Assessing Inter‐Rater Reliability and Trigger Performance of the Global Trigger Tool,” International Journal for Quality in Health Care 22, no. 4 (2010): 266–274, 10.1093/intqhc/mzq026. 20534607

[33] E. T. Deilkås , M. Haugen , M. B. Risberg , et al., “Longitudinal Rates of Hospital Adverse Events That Contributed to Death in Norway and Sweden From 2013 to 2018,” Journal of Patient Safety and Risk Management 26, no. 4 (2021): 153–160, 10.1177/25160435211026125.

[34] World Health Organization , “Conceptual fFamework for the International Classification for Patient Safety Version 1.1: Final Technical Report January 2009.” Geneva: WHO (2010). Contract No.: WHO/IER/PSP/2010.2.

[35] International JC , “International Patient Safety Goals: Joint Commission International,” (2025), https://www.jointcommission.org/en/standards/international-patient-safety-goals.

[36] World Health Organization , “Global Patient Safety Action Plan 2021–2030: Towards Eliminating Avoidable Harm in Health Care., WHO (2021).

[37] E. Von Elm , D. G. Altman , M. Egger , S. J. Pocock , P. C. Gøtzsche , and J. P. Vandenbroucke , “The Strengthening the Reporting of Observational Studies in Epidemiology (STROBE) Statement: Guidelines for Reporting Observational Studies,” Annals of Internal Medicine 147, no. 8 (2007): 573–577, 10.1097/EDE.0b013e3181577511. 17938396

[38] J. M. Aranaz‐Andrés , C. Aibar‐Remón , R. Limón‐Ramírez , et al., “Prevalence of Adverse Events in the Hospitals of Five Latin American Countries: Results of the ‘Iberoamerican Study of Adverse Events’ (IBEAS),” BMJ Quality & Safety 20, no. 12 (2011): 1043–1051, 10.1136/bmjqs.2011.051284. 21712370

[39] A. T. Dotta , L. E. Duarte Sotelo , M. A. Biaggioni , et al., “Detection of Adverse Events in Patients Interned in Medical Clinic Using the Global Trigger Tool,” Medicina 84, no. 1 (2024): 87–95. 38271935

[40] S. M. Moraes , T. C. A. Ferrari , N. M. P. Figueiredo , et al., “Assessment of the Reliability of the IHI Global Trigger Tool: New Perspectives From a Brazilian Study,” International Journal for Quality in Health Care 33, no. 1 (2021): mzab039, 10.1093/intqhc/mzab039. 33676370

[41] A. Mortaro , F. Moretti , D. Pascu , et al., “Adverse Events Detection Through Global Trigger Tool Methodology: Results From a 5‐Year Study in an Italian Hospital and Opportunities to Improve Interrater Reliability,” Journal of Patient Safety 17, no. 6 (2021): 451–457, 10.1097/PTS.0000000000000381. 28598897

[42] L. Pierdevara , A. M. Porcel‐Gálvez , A. M. Ferreira da Silva , S. Barrientos Trigo , and M. Eiras , “Translation, Cross‐Cultural Adaptation, and Measurement Properties of the Portuguese Version of the Global Trigger Tool for Adverse Events,” Therapeutics and Clinical Risk Management 16 (2020): 1175–1183, 10.2147/TCRM.S282294. 33299318 PMC7721282

[43] N. Grossmann , F. Gratwohl , S. N. Musy , N. M. Nielen , J. Donzé , and M. Simon , “Describing Adverse Events in Medical Inpatients Using the Global Trigger Tool,” Swiss Medical Weekly 149 (2019): w20149, 10.4414/smw.2019.20149. 31707720

[44] N. Marsh , E. N. Larsen , A. J. Ullman , et al., “Peripheral Intravenous Catheter Infection and Failure: A Systematic Review and Meta‐Analysis,” International Journal of Nursing Studies 151 (2024): 104673, 10.1016/j.ijnurstu.2023.104673. 38142634

[45] L. Sommella , C. De Waure , A. M. Ferriero , et al., “The Incidence of Adverse Events in an Italian Acute Care Hospital: Findings of a Two‐Stage Method in a Retrospective Cohort Study,” BMC Health Services Research 14 (2014): 358, 10.1186/1472-6963-14-358. 25164708 PMC4155122

