# Supplementary material for: Prevalence of Adverse Events in Mexico Using the Institute for Healthcare Improvement—Global Trigger Tool Method: A Retrospective Study
Source: J Eval Clin Pract. 2026 Mar 19;32(2):e70405. doi: 10.1111/jep.70405 (PMC13002140; doi:10.1111/jep.70405)
Supplement: Supplementary file 2 — Supplementary Table S2: Characteristics of hospital discharges in Hospital A. [file JEP-32-0-s002.docx]

**Supplementary Table 2.** Characteristics of hospital discharges in Hospital A

|  | **2022** | | | | | | **2023** | | | | | | |  |
| --- | --- | --- | --- | --- | --- | --- | --- | --- | --- | --- | --- | --- | --- | --- |
| **Clasification of discharges** | **July** | **August** | **September** | **October** | **November** | **December** | **January** | **February** | **March** | **April** | **May** | **June** | **TOTAL** | |
| Total hospital discharges | 834 | 841 | 821 | 852 | 803 | 933 | 854 | 662 | 851 | 815 | 937 | 850 | 10,053 | |
| Paediatric discharges <18 years | 164 | 173 | 190 | 186 | 217 | 220 | 169 | 154 | 219 | 176 | 237 | 212 | 2,317 | |
| Obstetric discharges | 191 | 206 | 172 | 215 | 182 | 244 | 249 | 155 | 217 | 203 | 227 | 198 | 2,459 | |
| Patients ≥18 years with length of stay < 24 hours | 26 | 20 | 24 | 36 | 19 | 25 | 34 | 41 | 34 | 35 | 41 | 64 | 399 | |
| Patients ≥18 years with length of stay ≥ 24 hours | **453*** | 442 | 435 | **415*** | 385 | 444 | **402*** | 312 | 379 | **401*** | 432 | 376 | 4,876 | |

*Bold numbers indicates the medical records of discharges included in the study
