# Supplementary material for: Prevalence of Adverse Events in Mexico Using the Institute for Healthcare Improvement—Global Trigger Tool Method: A Retrospective Study
Source: J Eval Clin Pract. 2026 Mar 19;32(2):e70405. doi: 10.1111/jep.70405 (PMC13002140; doi:10.1111/jep.70405)
Supplement: Supplementary file 3 — Supplementary Table S3: Characteristics of hospital discharges in Hospital B. [file JEP-32-0-s003.docx]

**Supplementary Table 3.** Characteristics of hospital discharges in Hospital B

|  | **2022** | | | | | | **2023** | | | | | |  | |
| --- | --- | --- | --- | --- | --- | --- | --- | --- | --- | --- | --- | --- | --- | --- |
| **Clasification of discharges** | **July** | **August** | **September** | **October** | **November** | **December** | **January** | **February** | **March** | **April** | **May** | **June** | | **TOTAL** |
| Total hospital discharges | 341 | 389 | 347 | 428 | 440 | 401 | 408 | 380 | 436 | 413 | 458 | 464 | | 4,905 |
| Paediatric discharges <18 years | 39 | 62 | 56 | 78 | 81 | 65 | 57 | 39 | 80 | 72 | 67 | 60 | | 756 |
| Obstetric discharges | 58 | 53 | 46 | 69 | 54 | 63 | 61 | 54 | 51 | 52 | 67 | 66 | | 694 |
| Patients ≥18 years with length of stay < 24 hours | 74 | 87 | 75 | 98 | 104 | 86 | 75 | 85 | 103 | 85 | 123 | 90 | | 1,085 |
| Patients ≥18 years with length of stay ≥ 24 hours | **170*** | 187 | 170 | **183*** | 201 | 187 | **215*** | 202 | 202 | **204*** | 201 | 248 | | 2,370 |

*Bold numbers indicates the medical records of discharges included in the study
