# Supplementary material for: Prevalence of Adverse Events in Mexico Using the Institute for Healthcare Improvement—Global Trigger Tool Method: A Retrospective Study
Source: J Eval Clin Pract. 2026 Mar 19;32(2):e70405. doi: 10.1111/jep.70405 (PMC13002140; doi:10.1111/jep.70405)
Supplement: Supplementary file 4 — Supplementary Table S4: Characteristics of hospital discharges in Hospital C. [file JEP-32-0-s006.docx]

**Supplementary Table 4.** Characteristics of hospital discharges in Hospital C

|  | 2022 | | | | | | 2023 | | | | | |  |
| --- | --- | --- | --- | --- | --- | --- | --- | --- | --- | --- | --- | --- | --- |
| **Clasification of discharges** | **July** | **August** | **September** | **October** | **November** | **December** | **January** | **February** | **March** | **April** | **May** | **June** | **TOTAL** |
| Total hospital discharges | 436 | 492 | 465 | 506 | 578 | 502 | 466 | 486 | 465 | 443 | 463 | 529 | 5,831 |
| Paediatric discharges <18 years | 37 | 44 | 52 | 58 | 85 | 60 | 43 | 43 | 38 | 39 | 44 | 51 | 594 |
| Obstetric  Discharges | 39 | 38 | 34 | 39 | 45 | 43 | 52 | 30 | 31 | 42 | 43 | 38 | 474 |
| Patients ≥18 years with length of stay < 24 hours | 95 | 119 | 156 | 130 | 178 | 124 | 99 | 195 | 117 | 133 | 110 | 180 | 1,636 |
| Patients ≥18 years with length of stay ≥ 24 hours | **265*** | 291 | 223 | **279*** | 270 | 275 | **272*** | 218 | 279 | **229*** | 266 | 260 | 3,127 |

*Bold numbers indicates the medical records of discharges included in the study
