# Supplementary material for: Prevalence of Adverse Events in Mexico Using the Institute for Healthcare Improvement—Global Trigger Tool Method: A Retrospective Study
Source: J Eval Clin Pract. 2026 Mar 19;32(2):e70405. doi: 10.1111/jep.70405 (PMC13002140; doi:10.1111/jep.70405)
Supplement: Supplementary file 5 — Supplementary Table S5: Triggers used from the Institute for Healthcare Improvement Global Trigger Tool (IHI‐GTT) for Measuring Adverse Events (1). [file JEP-32-0-s001.docx]

**Supplementary Table 5**. Triggers used from the Institute for Healthcare Improvement Global Trigger Tool (IHI-GTT) for Measuring Adverse Events (1).

| Cares Module Triggers | | Medication Module Triggers | |
| --- | --- | --- | --- |
| C1 | Transfusion or use of blood products | M1 | *Clostridium difficile* positive stool |
| C2 | Code/arrest/rapid response team | M2 | Partal thromboplastin time greater thn 100 seconds |
| C3 | Acute dialysis | M3 | International Normalized Ratio (INR) greater than 6 |
| C4 | Positive blood culture | M4 | Glucose less than 50 mg/dl |
| C5 | X-ray or Doppler studies for emboli or DVT | M5 | Rising BUN or serum creatinine greater than 2 times baseline |
| C6 | Decrease of greater than 25% in hemoglobin or hematocrit | M6 | Vitamin K administration |
| C7 | Patient Fall | M7 | Benadyl (Diphenhydramine) use Avapena |
| C8 | Pressure ulcers | M8 | Romazicon (Flumazenil) use |
| C9 | Readmission within 30 ays | M9 | Naloxone (Narcan) use |
| C10 | Restraint use | M10 | Anti-emetic use |
| C11 | Healthcare-associated infection | M11 | Over-sedation/hypotension |
| C12 | In-hospital stroke | M12 | Abrupt medication stop |
| C13 | Transfer to higher level of care | M13 | Other |
| C14 | Any Procedure complication |  |  |
| C15 | Other |  |  |
| Surgical Module Triggers | | Intensive Care Module Triggers | |
| S1 | Return to surgery | I1 | Pneumonia onset |
| S2 | Change in procedure | I2 | Readmission to intensive care |
| S3 | Admission to intensive care post-op | I3 | In-unit procedure |
| S4 | Intubation/reintubation/BiPap in Post Anesthesia Care Unit (PACU) | I4 | Intubation/reintubation |
| S5 | X-ray intra-op or in PACU |  |  |
| S6 | Inta-op or post-op death | Emergency Department Module Triggers | |
| S7 | Mechanical ventilation greater than 24 hours post-op | E1 | Readmission to ED within 48 hours |
| S8 | Intra-op epinephrine, norepinephrine, naloxone, or romazicon | E1 | Time in ED greater than 6 hours |
| S9 | Post-op troponin level greater than 1.5 ng/ml |  |  |
| S10 | Injury, repair, or removal of organ |  |  |
| S11 | Any operative complication |  |  |

**Reference:**

1.- Griffin Griffin FA, Resar RK. IHI Global Trigger Tool for Measuring Adverse Events (Second Edition). IHI Innovation Series white paper. Cambridge, Massachusetts: Institute for Healthcare Improvement; 2009. (Available on [www.IHI.org](http://www.IHI.org))
