# Supplementary material for: Prevalence of Adverse Events in Mexico Using the Institute for Healthcare Improvement—Global Trigger Tool Method: A Retrospective Study
Source: J Eval Clin Pract. 2026 Mar 19;32(2):e70405. doi: 10.1111/jep.70405 (PMC13002140; doi:10.1111/jep.70405)
Supplement: Supplementary file 6 — Supplementary Table S6: Categories according to the Institute for Healthcare Improvement Global Trigger Tool (IHI‐GTT) (1). [file JEP-32-0-s005.docx]

**Supplemetary Table 6.** Categories according to the Institute for Healthcare Improvement Global Trigger Tool (IHI-GTT) (1).

| **Category** | **Definition** |
| --- | --- |
| E | Temporary harm to the patient and required intervention |
| F | Temporary harm to the patient and requiered initial or prolonged hospitalisation |
| G | Permanent patient harm |
| H | Intervention required to sustain life |
| I | Patient death |

* The IHI-GTT categories, are based on the National Coordinated Council for Medicaition Error Reporting and Prevention (NCCMERP) Index, to classify the severity of adverse events (AEs)

**Reference:**

1.- Griffin FA, Resar RK. IHI Global Trigger Tool for Measuring Adverse Events (Second Edition). IHI Innovation Series white paper. Cambridge, Massachusetts: Institute for Healthcare Improvement; 2009. (Available on [www.IHI.org](http://www.IHI.org))
